# Misdiagnosis of HIV With Toxoplasmosis Encephalopathy With Progressive Memory Loss as the Initial Symptom: A Case Report

**DOI:** 10.3389/fneur.2022.809811

**Published:** 2022-03-15

**Authors:** Jingjing Wu, Xiumei Luo, Nanqu Huang, Yuanyuan Li, Yong Luo

**Affiliations:** ^1^Medical College of Soochow University, Suzhou, China; ^2^Department of Neurology, Third Affiliated Hospital of Zunyi Medical University (The First People's Hospital of Zunyi), Zunyi, China; ^3^National Drug Clinical Trial Institution, Third Affiliated Hospital of Zunyi Medical University (The First People's Hospital of Zunyi), Zunyi, China

**Keywords:** toxoplasmosis, toxoplasma encephalopathy, AIDS, memory loss, misdiagnosis

## Abstract

Toxoplasmosis encephalopathy (TE) is a kind of encephalopathy parasitic disease caused by *Toxoplasma gondii*. It is the most common opportunistic for central system infection in patients with acquired immunodeficiency syndrome (AIDS) or human immunodeficiency virus. Without early diagnosis and proper treatment, this opportunistic infection can be life-threatening. The common clinical manifestations of the disease include altered mental state, epilepsy, cranial nerve damage, paresthesia, cerebellar signs, meningitis, motor disorders, and neuropsychiatry. The most common presentation in about 75% of cases is a subacute episode of focal neurological abnormalities such as hemiplegia, personality changes, or aphasia. Imaging needs to be differentiated from multiple sclerosis, lymphoma, and metastases. We report a case of acquired immune deficiency syndrome complicated with toxoplasma encephalopathy with rapid progressive memory loss as the initial symptom and misdiagnosed as multiple sclerosis. Through the comprehensive analysis of the clinical symptoms and imaging examination of this disease, we hope to enhance the confidence of clinicians in the diagnosis of this disease.

## Introduction

*Toxoplasma gondii* is a parasite that spreads widely around the world, affecting more than a third of the world's population (1). In healthy, immuno-functioning hosts, acute infection progresses to asymptomatic latent infection, in which the parasite encysts in the heart, skeletal muscle, brain parenchyma, and retina with no or mild symptoms. However, when there is a problem with immune function, such as after an immune deficiency or organ transplant or chemotherapy in cancer patients, latent infection can be reactivated and latent bradyzoites transformed into rapidly replicating tachyzoites. If no intervention is taken at this time, the consequences can be severe and life-threatening, with mortality rates up to 100% (1, 2).

AIDS remains an important public health problem in China despite the tremendous efforts made by the Chinese government over the past 30 years (3, 4). Since HIV was first reported and noticed in China, the prevalence of HIV has changed. In the past 10 years, the incidence of AIDS has increased significantly, but it has been well controlled in the provinces with high incidence and among young and middle-aged people. However, the incidence of the disease has increased in some provinces and among people over the age of 55, which deserves our attention (5). Opportunistic infection caused by parasitic infection is one of the most common causes of morbidity and mortality of AIDS patients, among which *T. gondii* infection is a very important parasitic infection (6). About 40% of studies reported so far in South America and the Asian continent have found toxoplasma latent infection rates as high as 41.9-72% in HIV-infected people (7).

About 30-40% of HIV-infected individuals with *T. gondii* will develop Toxoplasmosis encephalopathy (TE) (8). Nissapaton et al. (9) reported a serum survey of 505 AIDS patients and found that 226 (44.8%) were infected with Toxoplasma, of which 27 had toxoplasma encephalitis. Patients with TE presented with focal neurological dysfunction and signs. Usually, 58.89% of patients presented with subacute onset. Between 15 and 25% of patients present with acute aggression, presenting as sudden seizures or bleeding. Mild hemiplegia and/or language abnormalities are the most common initial symptoms. Headaches, mental changes, lethargy, and brain stem and cerebellar disorders have also been reported (10, 11).

However, reports of TE with progressive memory loss as the initial symptom are rare. It is also difficult to diagnose TE. Due to insufficient understanding of HIV, people often hide their medical history, and it is difficult to diagnose TE by imaging alone. The imaging manifestations of TE are very similar to other encephalopathies, such as brain abscess, metastatic tumor, and central nervous system lymphoma (10). Primary hospitals have limited means of examination, and the serum and cerebrospinal fluid tests for parasites need to be completed by delivery companies. The results will be delayed, so it is easy to misdiagnose and miss diagnosis. Therefore, it is very important to broaden clinical thinking. This paper mainly discusses how to detect and diagnose HIV infection complicated with TE as early as possible and how to treat it early.

## Case Report

On September 25, 2017, a 33-year-old rural woman was admitted to the Department of Neurology of The First People's Hospital of Zunyi city due to “progressive memory decline for half a year and left upper limb weakness for 2 weeks.”

Since March 2017, the patient has developed progressive memory decline without obvious causes, which is manifested as scatterbrain (often unable to find things), decreased calculation ability (wrong calculation when shopping), and gradually aggravated. Later, the patient could not find his home, accompanied by slow reaction and slow walking, and did not pay attention to it. On May 20, 2017, there was no obvious cause of headache, blurred vision and decreased vision, which was slightly better after traditional Chinese medicine (TCM) acupuncture treatment in another hospital (the specific diagnosis and treatment process is unknown). On or about September 10, 2017, the patient developed weakness and numbness in the distal fingers of the left upper limb, and the unstable holding of the left upper limb, without convulsions, disturbance of consciousness and fever, no vomiting, difficulty swallowing, dysphagia and diplopia. Past medical history: The patient had suffered a head trauma 10 years earlier; 2 years ago, she was treated for viral herpes in another hospital and got better. Later, she developed local itching after sun exposure. The patient denied any history of drug use, unclean sexual behavior, or blood product use.

### Auxiliary Examination Prior to Admission

**Figure 1:**
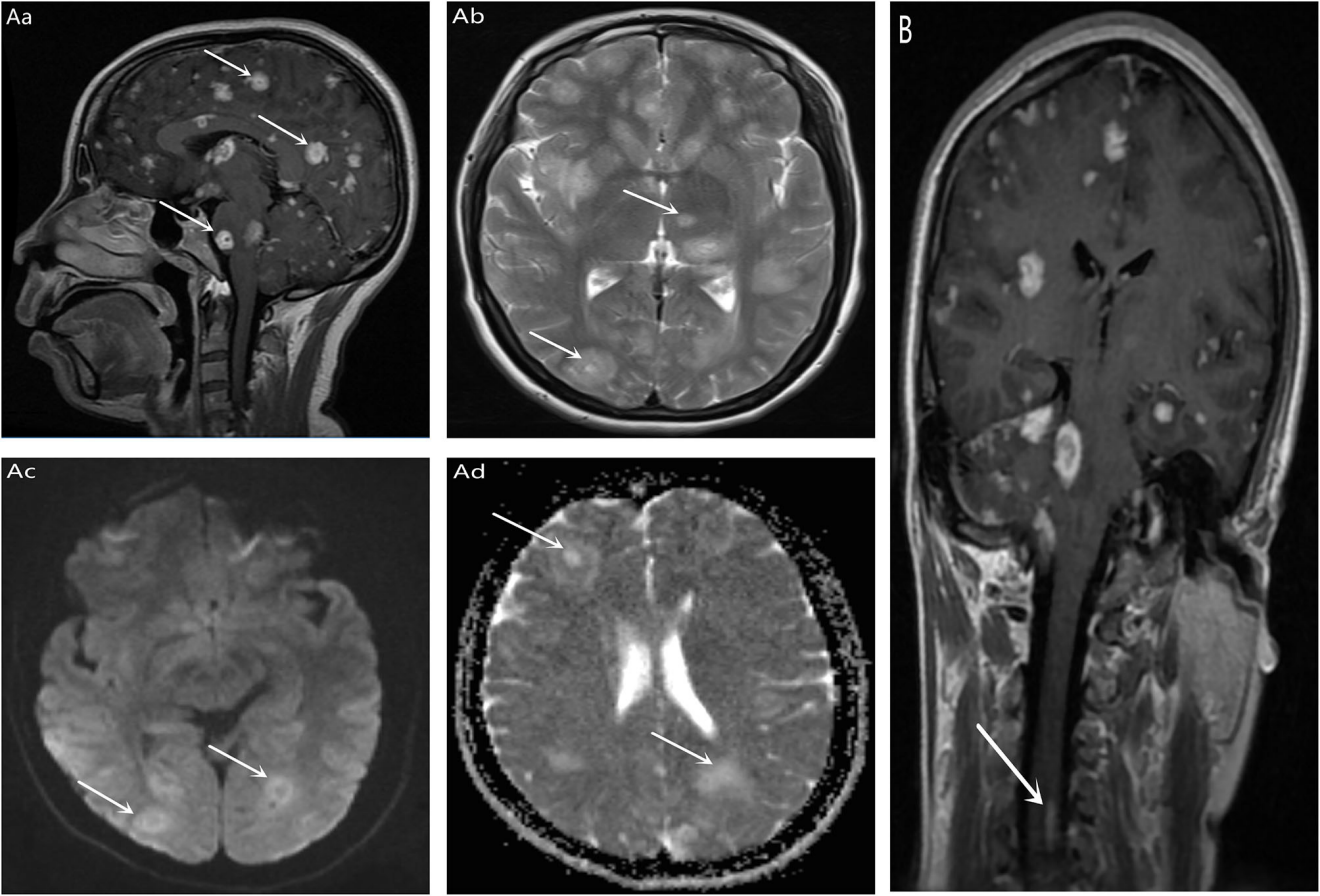
T1 **(Aa)** and T2 **(Ab)** signal of multiple Clumps long T1 in bilateral brain, cerebellum, brainstem, and corpus callosum. DWI **(Ac)** and ADC **(Ad)** showed high signal (indicated by white arrows in the figure); Enhanced scan showed bilateral nodules, cerebellum and brainstem multiple nodular, annular enhancement, the largest diameter of about 18 mm (indicated by the white arrow in the figure). **(B)** No separate MRI of the spinal cord was performed, and there was also an unexpected high signal in the spinal cord during cranial enhancement (indicated by the white arrow in the figure).

Follow the MRI scan of the Affiliated Hospital of Zunyi Medical University for cranial MRI + MRA: Multiple lesions of the brain, cerebellum, brainstem and corpus callosum were found in both sides (2017-9-21, Figure 1A) are likely to be acute disseminated encephalomyelitis. However, infectious lesions and metastatic tumors still need to be considered, and an enhanced scan is recommended. Partial cavitation sella bilateral maxillary sinus and ethmoid mucosa thickening; MRA shows the right anterior cerebral artery is smaller and slimmer. Enhanced cranial magnetic resonance (2017-9-21): Multiple bilateral lesions of the brain, cerebellum, and brainstem were considered (Figure 1A), and small spinal cord enhancement lesions were considered (Figure 1B). Multiple sclerosis was diagnosed based on history and general examination. Hormonal treatment was ineffective, and then the patient was referred to our hospital.

### Physical Examination at Admission

T 36.1°C, P 70 bpm, R 18 bpm, Bp 114/82 mmHg, clouding of consciousness, smooth language, slow response, memory impairment, normal judgment and timing orientation, poor eye movement, grade 5 muscle strength in the right upper limb, 4 muscle strength in the left upper limb, grade 3 muscle strength in the lower limbs, normal muscle tension, tendon reflexes (++), pathological signs were not elicited, meningeal irritation was negative.

### Auxiliary Examination After Admission

pulmonary CT: grinding glass in the left lower lobe, considering inflammation; ESR: 69.7 mm/h, abnormal detection of cerebrospinal fluid (Table 1), abnormal blood electrolyte (Table 2), blood lipid: TG 6.06 mmol/L, high density lipoprotein 0.55 mmol/L, low density lipoprotein 2.01 mmol/L, apolipoprotein A 0.76 g/L. Fasting blood glucose, myocardial enzymes, liver function, renal function, blood routine, AFP, tuberculosis antibody IgG, rheumatoid factor, thyroid function, HCG, lung cancer tumor markers, parathyroid hormone, urine routine, coagulation function, troponin I, all the above inspection items was not abnormal; in total abdominal CT, cardiac ultrasound, electrocardiogram, and abdominal ultrasound were no abnormalities at all. A diagnosis of multiple sclerosis was made based on history and general examination, but hormone treatment in other hospitals was ineffective.

**Table 1:** Results of cerebrospinal fluid test.

**Cerebrospinal fluid** **testing item**	**2017-9-26**	**2017-9-28**	**Reference value**
Turbidity	clear and transparent	clear and transparent	clear and transparent
Color	Colorless	Colorless	Colorless
Clot	without clot	without clot	without clot
CSF pressure	330 mmH_2_O	Untested	70-180 mmH_2_O
Total cell number	20^*^10^6^/L	44^*^10^6^/L	-
WBC	18^*^10^6^/L	32^*^10^6^/L	0-8
Monocytes number	16^*^10^6^/L	28^*^10^6^/L	<10^*^10^6^/L
Number of multinucleated cells	2^*^10^6^/L	4^*^10^6^/L	-
Chlorine	126.1 mmol/L	127.1 mmol/L	120-130mmol/L
Glucose	2.4 mmol/L	2.1 mmol/L	2.5-4.4 mmol/L
ADA	2.4 U/L	4.0 U/L	0-3 U/L
Protein	2.03 g/L	2.04 g/L	0-0.45 g/L
Lactate dehydrogenase	38.6 U/L	59.6 U/L	10-25 U/L
Acid-fast dyeing	No acid-fast bacillus found	No acid-fast bacillus found	No acid-fast bacillus found
Ink dyeing	Not found Cryptococcus neoformans	Not found Cryptococcus neoformans	Not found Cryptococcus neoformans
Exfoliated cells	Untested	see a small number of lymphocytes	- -
Cerebrospinal fluid culture	72 h of sterile growth	72 h of sterile growth	-

The *symbol indicates multiply.

**Table 2:** Results of blood ion tests.

**Blood plasma examination item**	**2017-09-25**	**2017-09-28**	**2017-09-29**	**Reference interval**
Potassium	3.5 mmol/L	3.8 mmol/L	3.4 mmol/L	3.5-5.5 mmol/L
Sodium	132.1 mmol/L	132.8 mmol/L	133.2 mmol/L	135-145 mmol/L
Chlorine	104.1 mmol/L	104.6 mmol/L	102.4 mmol/L	98-111 mmol/L
Calcium	2.27 mmol/L	2.16 mmol/L	2.10 mmol/L	2.25-2.58 mmol/L
Osmotic pressure	284.2 mmol/L	285.6 mmol/L	286.4 mmol/L	0.97-1.16 mmol/L
CO2	20.6 mol/L	19.4 mol/L	17.9 mol/L	22-32 mol/L

Our hospital usually takes HIV test as one of the routine examinations, and the patient was found to be HIV positive several days after admission. Although the patient had denied any history of drug abuse, unclean sex or blood product use when admitted, we still cannot exclude whether the patient and his family members have hidden medical history. At this point, we need to consider a central nervous system infection. Combined with the results of cerebrospinal fluid examination, there is insufficient evidence for viral infection (high cerebral pressure), tuberculosis infection (no history of tuberculosis infection and obvious wasting symptoms, negative acid-fast staining) and cryptococcus infection (negative ink staining). We then sent the patient's blood to Zunyi CDC(The Centers for Disease Contro, CDC) for a retest of HIV antibody test, and sent cerebrospinal fluid and blood to Jinyu Center for parasite test.

On September 28, 2017, Zunyi CDC and Prevention HIV antibody test positive Jinyu testing center for cerebrospinal fluid and blood parasite test results showed that Toxoplasma IgG antibody positive diagnosis consideration:1 AIDS; 2 TE; We treated with dehydration of mannitol and glycerin fructose to reduce intracranial pressure, and sulfamethoxazole trimethoprazole (smz-tmp) 1.44 g/time orally, 3 times/day, combined with clindamycin 600 mg/time intravenously, every 6h1 times according to the expert consensus (“Expert consensus on clinical diagnosis and treatment of AIDS complicated with Toxoplasma encephalitis”). Radiographs were to be reassessed after 6 weeks of regular treatment, but the patient and his family refused further treatment and discharged from the hospital after several days of treatment.

## Discussion

In this case, a young female patient presented with rapid progressive memory loss as the first symptom, but the patient and her family did not notice it. The main symptoms are headache, blurred vision and weakness. Brain MRI showed a “starry sky” appearance, and enhanced MRI showed multiple nodular enhancement lesions in brain, cerebellum, brainstem and corpus callosum (Figure 1A), and small focal enhancement lesions in cervical spinal cord (Figure 1B). Overall, initial diagnosis of multiple sclerosis is reasonable for the following reasons: (1) Gender, age, and clinical symptoms support, Multiple sclerosis is the most common inflammatory nervous system disease in young people, it is a potentially serious cause of neurological dysfunction throughout the adult population (12). (2) In the course of the disease, there is a suspicious remission process after TCM treatment, most patients may present with recurrent remission of recurrent neurological symptoms (12). (3) MRI showed obvious symptoms of cervical spinal cord (consistent with spatial multiple), on conventional MRI imaging, 90% of multiple sclerosis patients will have spinal cord injury once onset (13), and 30-40% of patients will have spinal cord injury before onset or symptoms (14). Spinal cord lesions are part of the diagnostic criteria for spatial spread in the International Panel guidelines (15). But ultimately, we don't consider Multiple sclerosis and here's the reasons: (1) The overall course of disease continues to be one-way progression, Although the patient showed cognitive impairment with rapid memory loss, there were no obvious neurological manifestations common to multiple sclerosis, such as optic neuritis, diplopia, sensory loss, limb weakness, gait ataxia, and loss of bladder control (12). (2) Magnetic resonance imaging is inconsistent with classical MS results. (3) Cerebrospinal fluid pressure has obvious changes. However, there is no specificity in the changes of cerebro spinal fluid (CSF) of TE. Chinese studies have shown that about 42.6% of AIDS/TE patients can have increased CSF pressure, and 66.0% of PATIENTS can have increased CSF protein content (16). (4) Symptoms of multiple sclerosis did not improve after treatment. TE is eventually diagnosed by a combination of HIV antibody positivity, CSF and plasma IgG antibody positivity. New epidemiological evidence based on a meta-analysis of 74 studies of toxoplasma and HIV coinfection in 34 countries shows a combined serum prevalence of 35.8% worldwide. The prevalence in Asia and the Pacific was 25.1% and, as expected, populations in developing countries showed higher comorbidities (54.7%) compared with middle-income countries (34.2%) and high-income countries (26.3%). As developing countries, we should pay more attention to the diagnosis and treatment of the disease. Next, we will discuss the problems that should be paid attention to in the diagnosis of this case.

### Relationship Between HIV-Infected Toxoplasma Encephalopathy and Memory Loss

Toxoplasma titer is generally associated with poorer cognitive performance (17). A 2010 study by Fekadu (18) showed that such cognitive decline caused by toxoplasma seropositivity is particularly obvious in low-income people (19) and people with low education level (20). However, while many studies have shown that tracking toxoplasma seropositivity is associated with poorer cognitive function, other studies have shown that positive seropositivity is not entirely harmful. In a 2017 study by Wyman et al., Toxoplasma antibody titers in toxoplasma infection sero-positive participants were negatively correlated with memory (21). This is contrary to our understanding that the higher the antibody titer is, the more serious the cognitive impairment is.

Studies have shown that almost half of people living with HIV have mild to moderate neurocognitive impairment (22, 23). Since both HIV and Toxoplasma infection affect cognitive function, could the combination of the two diseases make cognitive impairment worse? It is well known that there are many reasons affecting cognitive impairment in HIV patients, such as age, drug abuse, chronic hepatitis C, etc. (24). However, the effect of latent *T. gondii* (LT) on NC has not attracted enough attention. In a 2016 study (25) of nearly 250 adults with HIV+ and HIV–, found that underlying toxoplasmosis was associated with poorer overall cognitive performance. Neurocognitive impairment (NCI) was very common among HIV+ patients (36.5 vs. 11.7%; *p* < 0.001). Most HIV+ patients had mild NCI (29.21.6%) and the rest had moderate NCI. In HIV-infected patients, lower levels of Toxoplasma IgG were associated with poorer cognitive performance, contrary to what we expected. Perhaps because toxoplasma levels decline over time, it is possible that toxoplasma negatively affects cognition due to the slow and cumulative effects of infection rather than the acute effects (which are associated with higher levels) (26, 27). This is consistent with the results of the 2017 study by Wyman et al. (21), which is worth thinking about and points to the direction of further research.

The patient was a young patient with rapid progressive memory loss. It is not clear whether HIV or Toxoplasma is the cause, but the combination of the two diseases is not likely, and some confounding factors, such as smoking, alcohol consumption, education level, family environment and psychological factors, should also be considered. At the same time, it also suggests that HIV with toxoplasma infection should be considered as one of the differential diagnoses in young patients with rapid progressive memory loss in addition to some common diseases.

### A Brief Discussion on the Differential Diagnosis of TE on Imaging

In this case, MRI showed multiple ring enhancement lesions. Can multiple sclerosis be considered in the initial diagnosis of this case? The presence of annular lesions in clinical isolation syndrome and early multiple sclerosis disease may have implications for future studies of disease activity and progression, Blindenbacher et al. (28) reported in the 2020 study. However, the ring signs mentioned above mainly appear on SWI of MRI, this is different from the annular appearance in this case.

So, what should be considered in the differential diagnosis of annular lesions? The differential diagnosis of ring enhancement is very extensive. In non-immunocompromised patients, the most common diseases were metastatic disease and septic emboli, and in immunocompromised patients lymphoma was the most common (29). Metastatic disease usually has a definite history of tumor and is of equal size, usually at or near the gray-white matter junction. Emboli usually result in acute infarction of the associated vascular distribution, which is not seen in toxoplasmosis or metastatic disease. Lymphoma is usually larger than TE, more invasive in shape, heterogeneous in enhancement, and may spread to a limited extent (30, 31).

The case was definitively diagnosed as TE, the most classic and common MRI manifestation of toxoplasmosis is the enhanced T1W “eccentric target sign,” which consists of three alternating regions: the innermost eccentric enhanced core, the moderate-low signal region, and the peripheral high-signal enhanced edge. This appearance produces a ring of enhancement, and the presence of a central nodule is usually eccentric, hence the term “eccentric target.” Can TE be excluded if there is no eccentric target sign? Although such presentations are highly suggestive of toxoplasmosis, such presentations are found in only 30% of cases (32). Therefore, TE cannot be excluded even if there is no characteristic manifestation, and the patient's medical history, physical examination and serological results should be combined to assist the diagnosis.

### Analysis of Causes of Misdiagnosis

In this case, the patient presented with progressive memory loss as the first symptom and received little attention. This is a wake-up call for us to see such clinical manifestations in young patients without any vital events, and we should think about the presence of organic disease. The patient in this case may have hidden his medical history, so we should strengthen health education in the economically underdeveloped and relatively backward areas. At the same time, whether the examination of some infectious diseases should be included in the routine examination is worth our vigilance and consideration. If HIV infection is known, we need to be aware of the possibility of TE. A meta-analysis involving global studies found that the serum antibody (IgG) positive rate of *T. gondii* was significantly higher in AIDS patients than in healthy people (46.1 vs. 36.6%) (33). A recent systematic review of 111 studies from 37 countries reported a pooled incidence of 44.22% of Toxoplasma infection in AIDS patients, higher than that obtained based on IgM analysis (3.24%) and molecular methods (26.22%), highlighting the high incidence of Toxoplasma infection in AIDS patients (34). Belanger et al. (35) analyzed 116 AIDS patients with toxoplasmosis, including 103 cases (88.8%) of TE, 7 cases (6%) of Toxoplasmosis lung disease, 4 cases (3.5%) of toxoplasmosis, and 2 cases (1.7%) of disseminated toxoplasmosis. At the same time, we send check out by considering the patients' economic reasons only checked IgG antibody in serum and cerebrospinal fluid, did not check the IgM and the degree of antibody, check diagnose diseases for us both for the recent infection or reactivation of *T. gondii* and prognosis is meaningful, if conditions allow should try to improve the inspection. Of course, if the CD4+T lymphocyte count can be tested, and regular review after anti-toxoplasma treatment, it will be more conducive to our diagnosis. Due to the financial constraints of patients, we did not send out the examination of oligoclonal zone. The specific change of MS in CSF is marked by the detection of oligoclonal band (OCB). The absence of OCB has a high negative predictive value, indicating the presence of a danger signal on diagnostic tests, and other diagnoses should be considered in such patients (36), this is also important for our differential diagnosis.

Meanwhile, we considered the spatial multiple of MS when we saw lesions in cervical spinal cord on MRI, but ignored the involvement of spinal cord in TE. The most common clinical manifestation of toxoplasmosis in HIV infection is encephalitis, which usually occurs when the CD4+ cell count is below 200 cells/μl and generally follows the reactivation of latent infection (37). Spinal cord involvement can occur and manifest as motor or sensory impairment of the limbs, bladder or bowel dysfunction or both, and local pain, Therefore, numbness and weakness of the left upper limb in this patient should be related to spinal cord injury.

### Clinical Guiding Significance

TE can be diagnosed for AIDS patients by meeting the following three points: (1) Patient's cerebrospinal fluid or serum anti-Toxoplasma IgG and/or IgM antibody are positive; (2) Head magnetic resonance imaging (MRI) showed multiple nodular or round-shaped lesions, T1W1 Low signal, T2W2 hyperintensity, with edema zone around, T1W1 showed “target sign” enhancement; (3) Brain lesions absorbed by brain MRI lesion 2 weeks after diagnostic treatment (7). It should be noted that toxoplasma exists only intermittently in the spinal fluid. In this case, a single positive CSF result is lucky, but if no positive CSF result is found, the diagnosis should be confirmed by repeated tests. It has been proved that repeated samples can improve the sensitivity of the test.

Studies have shown that HIV/AIDS patients in Asia and Africa are more likely to be infected with Toxoplasma than those in the Americas and Europe (38), and that timely and sustained highly active antiretroviral therapy (HAART) and preventive therapy are important for HIV-positive patients, reducing TE risk by 50% on antiretroviral therapy and 53% on preventive therapy (39). Therefore, we recommend routine serological screening for Toxoplasma infection in HIV patients in endemic areas. Positive patients are at risk of infection reactivation, and negative patients should also be informed to prevent primary infection.

At the same time, we should also actively carry out health education, AIDS is usually high among young people, but some studies show that the coverage of AIDS health education reaches 80% in rural areas of western China, and attention should be paid to the elderly, poor people and minority communities (40). The aim is to establish routine serological monitoring, counseling, nursing and preventive treatment programs to prevent HIV infected with severe toxoplasma encephalopathy and, if infected, identify and treat them as soon as possible.

## Data Availability Statement

The original contributions presented in the study are included in the article/supplementary material, further inquiries can be directed to the corresponding author/s.

## Author Contributions

JW completed literature search and article writing. XL and YuL assisted in the collection of materials and related data and pictures. NH assisted in the collation of pictures and the modification of the article. YoL designed the ideas of the article and the modification of the article. All authors read and approved the final manuscript.

## Funding

This work was supported by Guizhou Province High-level Innovative Talent Cultivation Project (2015-25) and Zunyi City 15851 Talent Elite Project Project.

## Conflict of Interest

The authors declare that the research was conducted in the absence of any commercial or financial relationships that could be construed as a potential conflict of interest.

## Publisher's Note

All claims expressed in this article are solely those of the authors and do not necessarily represent those of their affiliated organizations, or those of the publisher, the editors and the reviewers. Any product that may be evaluated in this article, or claim that may be made by its manufacturer, is not guaranteed or endorsed by the publisher.
